# Patchy Vasoconstriction Versus Inflammation: A Debate in the Pathogenesis of High Altitude Pulmonary Edema

**DOI:** 10.7759/cureus.10371

**Published:** 2020-09-10

**Authors:** Rajan Sharma Kandel, Rohi Mishra, Jeevan Gautam, Amer Alaref, Abdallah Hassan, Nusrat Jahan

**Affiliations:** 1 Internal Medicine, California Institute of Behavioral Neurosciences & Psychology, Fairfield, USA; 2 Neurology, California Institute of Behavioral Neurosciences & Psychology, Fairfield, USA; 3 Diagnostic Radiology, California Institute of Behavioral Neurosciences & Psychology, Fairfield, USA; 4 Diagnostic Radiology, Thunder Bay Regional Health Sciences Centre, Thunder Bay, CAN; 5 Diagnostic Imaging, Northern Ontario School of Medicine, Sudbury, CAN; 6 Breast Imaging, Thunder Bay Regional Health Sciences Centre/Linda Buchan Centre, Thunder Bay, CAN

**Keywords:** altitude sickness, pathophysiology, patchy vasoconstriction, inflammation, genetics, high altitude pulmonary edema

## Abstract

High altitude pulmonary edema (HAPE) occurs in individuals rapidly ascending at altitudes greater than 2,500 m within one week of arrival. HAPE is characterized by orthopnea, breathlessness at rest, cough, and pink frothy sputum. Several mechanisms to describe the pathophysiology of HAPE have been proposed in different kinds of literature where most of the mechanisms are reported to be activated before a drop in oxygen saturation levels. The majority of the current studies favor diffuse hypoxic pulmonary vasoconstriction (HPV) as a pathophysiological basis for HAPE. However, some of the studies described inflammation in the lungs and genetic basis as the pathophysiology of HAPE. So, there is a major disagreement regarding the exact pathophysiology of HAPE in the current literature, which raises a question as to what is the exact pathophysiology of HAPE. So, we reviewed 23 different articles which include clinical trials, review articles, randomized controlled trials (RCTs), and original research published from 2010 to 2020 to find out widely accepted pathophysiology of HAPE. In our study, we found out sympathetic stimulation, reduced nitric oxide (NO) bioavailability, increased endothelin, increased pulmonary artery systolic pressure (PASP) resulting in diffuse HPV, and reduced reabsorption of interstitial fluid to be the most important determinants for the development of HAPE. Similarly, with the evaluation of the role of inflammatory mediators like C-reactive protein (CRP) and interleukin (IL-6), we found out that inflammation in the lungs seems to modulate but not cause the process of development of HAPE. Genetic basis as evidenced by increased transcription of certain gene products seems to be another promising hypoxic change leading to HAPE. However, comprehensive studies are still needed to decipher the pathophysiology of HAPE in greater detail.

## Introduction and background

High altitude pulmonary edema (HAPE), which is a non-cardiogenic pulmonary edema [1], usually occurs in rapidly ascending non-acclimatized healthy individuals above 2,500 to 3,000 meters [2, 3]. One-fifth of the earth’s surface is covered by mountains that attract many tourists yearly [4]. Military deployment worldwide in high-altitude (HA) areas and decreased time of acclimatization due to air transport also account for the increasing incidence of HAPE [5]. HAPE can develop within hours to days, typically within the first week after arrival at high altitude [3, 6]. It presents with weakness, dyspnea, and dry cough with exertion which progresses to dyspnea at rest, rales, cyanosis, and pink-frothy sputum [7]. HAPE is the most common cause of high-altitude related deaths [8], accounting for up to 50% mortality in untreated conditions [7]. HAPE is precipitated by a severe and abrupt change in barometric pressure [9]. Subsequently, decreased partial pressure of oxygen causes uneven vasoconstriction resulting in exaggerated capillary pressure, increased capillary permeability, and fluid collection in the lungs [9, 10]. Exposure to hypoxia increases pulmonary arterial systolic pressure (PASP) that is directly proportional to the degree of hypoxia [11] and altitude [12]. Apart from hypoxia, other factors like ventilatory control, activation of the sympathetic nervous system, endothelial function, reabsorption of sodium, and water from alveolar epithelium likely increase HAPE susceptibility [2].

Several mechanisms to describe the pathophysiology of HAPE have been proposed in different scientific works which creates a major disagreement regarding the exact pathophysiology of HAPE in the current literature. Many of the studies conducted in humans favor patchy vasoconstriction as a result of hypoxia and increased sympathetic activity with additional contribution from endothelin [13] combined with reduced bioavailability of endogenous vasodilator nitric oxide (NO) [14]. According to Swenson et al., high capillary pressure (confirmed by measurements in humans) precipitates a high-permeability non-inflammatory type lung edema called “capillary stress failure” [2]. This causes extravasation of proteins and erythrocytes into the alveolar space without inflammation [2]. However, an inflammatory basis in the pathogenesis of HAPE is another promising theory that is gaining limelight. Hilty et. al [15] proposed that the inflammation in the lungs causes HAPE where acute hypoxic stimulus, in turn, increases blood concentration of eicosanoids [16], C-reactive protein (CRP), tumor necrosis factor (TNF-a), interleukins (IL-1, IL-2, IL-6) [17, 18] and urinary concentration of eicosanoids [19]. In this study, they found higher levels of these inflammatory markers in acute mountain sickness developing patients in comparison to healthy control [15]. They also described an association between viral upper airway infection with HAPE susceptibility [15, 20, 21]. Recently much attention has been given to acquiring knowledge on the genetic basis of HAPE, where genome-wide association and candidate gene molecular approaches are commonly used [10]. When an individual has previous experience of HAPE, the prevalence of having such episodes in the future is highly increased (>60%) in high-altitudes indicating individual susceptibility and genetic basis of HAPE [6]. 

Currently, there is very little literature that points to the role of inflammatory mediators in the pathogenesis of HAPE. In this study, we aim to provide a comprehensive overview of the pathophysiology of HAPE from current literature. We identified 23 potential articles published within the last 10 years to gain insight into the pathological process occurring inside the lungs at high altitudes (HA). Further studies are required to be conducted at HA as well as in lab simulating hypobaric and hypoxic conditions encountered at HA to identify the exact pathophysiology of HAPE. Identifying the exact pathophysiology of HAPE will help formulate preventive and curative strategies for this life-threatening condition.

## Review

Method

**Figure 1:**
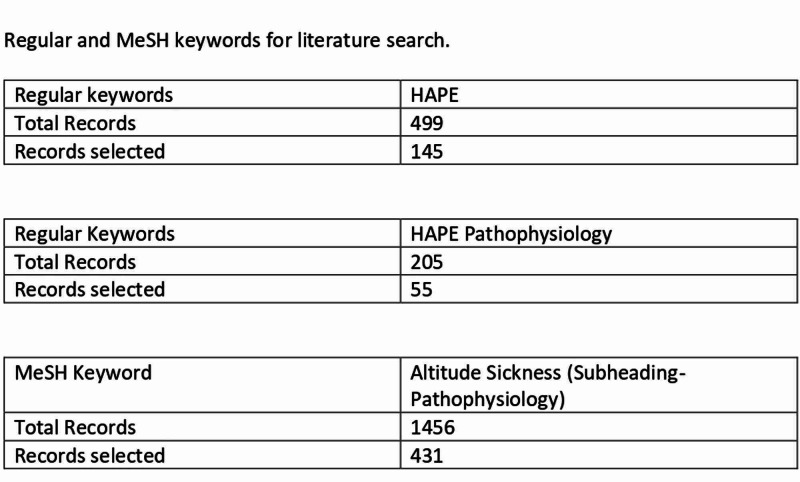
Regular keywords, MeSH Terms, and MeSH subheadings used for literature search MeSh: Medical Subject Headings

We used the PubMed database for data search and collection utilizing Medical Subject Headints (MeSH) terms, MeSH subheadings, and keywords. Figure 1 shows the keywords used for the literature search.

Full-text articles including clinical trials, reviews, original research, and randomized controlled trials (RCTs) involving human subjects and those articles published within the last 10 years in the English language were included for review. We excluded non-human studies, literature older than 10 years, and published in languages other than English.

Results

**Figure 2:**
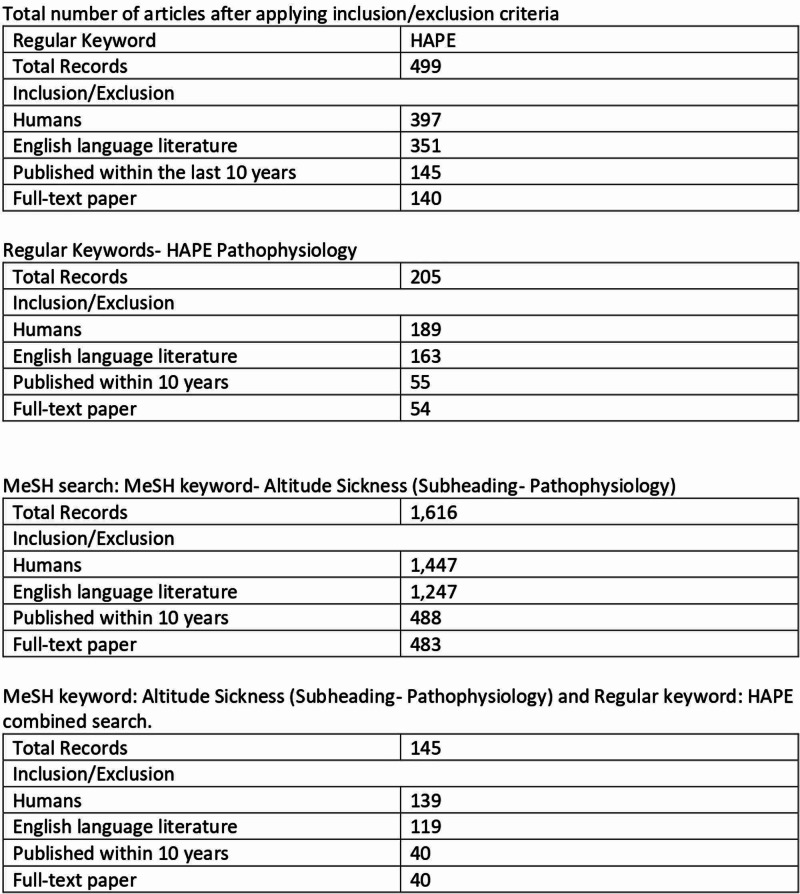
Application of inclusion/exclusion criteria MeSH: Medical Subject Headings

From 140 potential articles, a total of 100 articles from the keyword search “HAPE” were excluded due to either a lack of outcome of interest “Pathophysiology of HAPE” or duplication. The literature search identified 40 potentially relevant citations when inclusion and exclusion criteria were applied. All citations were from PubMed. After reviewing the title and abstracts, 23 articles were found relevant - 12 clinical trials, six review articles, three Randomized Controlled Trials (RCTs), and two original research. Figure 2 shows the details about the inclusion/exclusion criteria.

**Figure 3:**
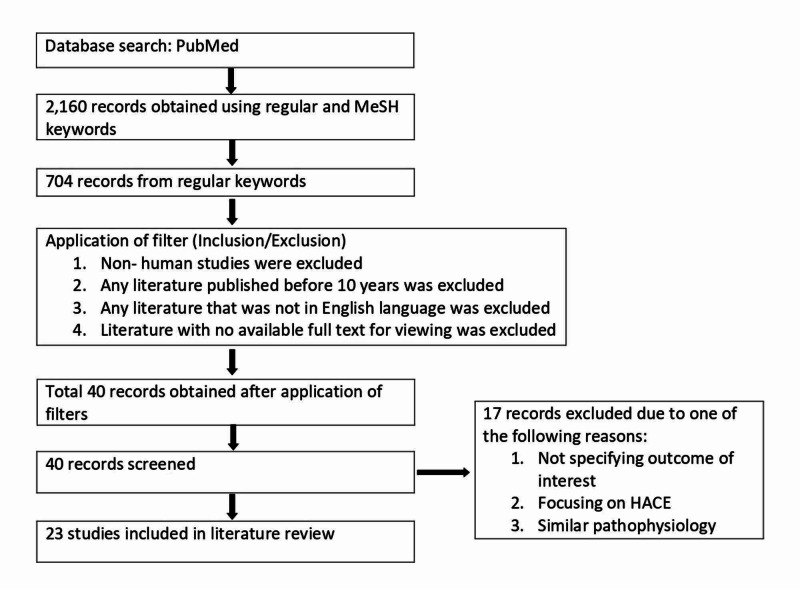
Selection process HACE: high altitude cerebral edema; MeSh: Medical Subject Headings

Figure 3 shows the process of article selection.

Discussion

In this analysis of 3,027 subjects, we examined pathophysiological changes in lungs at high altitude (HA). We analyzed 23 studies for our review article, where seven studies discussed mainly on hypoxic pulmonary vasoconstriction (HPV), four studies discussed mainly on inflammation, four studies discussed mainly on the genetic basis, and rest on the physiology of lungs at HA. Our study compared two distinct pathophysiologies: HPV and inflammation. Most of the literature favors HPV as a pathophysiological basis of high altitude pulmonary edema (HAPE) [2]. The present review article is aimed to provide a comprehensive overview of the pathophysiology of HAPE. Table 1 shows a brief discussion of the most relevant articles we reviewed.

**Table 1:** Brief findings of the most relevant articles. HAPE: high altitude pulmonary edema; TLC: total lung capacity,  FVC: functional vital capacity; HA: high altitude; PASP: pulmonary artery systolic pressure; NO: nitric oxide; HPV: hypoxic pulmonary vasoconstriction

First author, Year	Study design	Total number of subjects	Main points
Bailey, 2010 [14]	Clinical trial	26	This study suggested that increased vascular resistance and pulmonary artery systolic pressure (PASP) at HA decreases pulmonary NO bioavailability due to free radical formation.
Mounier, 2011 [22]	Clinical trial	16	Authors showed that predictive factor of HAPE to be PASP response in relation to hypoxia and mild exercise from basal normoxic resting values
Clarenbah, 2012 [23]	Research article	18	Individuals developing overt HAPE initially developed a greater fall in FVC compared to controls. Decreased inspiratory muscle force is correlated with reductions in FVC. Reduced lung compliance from the interstitial fluid collection in the lungs and inspiratory muscle weakness seem to be major factors for fall in FVC and TLC.
Swenson, 2012 [2]	Review	NA	Increased microvascular pressures due to a rise in pulmonary vascular resistance or HPV is a commonly accepted pathophysiology of HAPE. Dynamic changes in the permeability of the alveolar-capillary barrier induced by hydrostatic stress cause mechanical damage. This leads to proteins and erythrocyte leaks into alveolar space without signs of inflammation.
Korzeniewski, 2015 [24]	Review	NA	Non-homogenous HPV, an adaptive vasomotor response triggered by alveolar hypoxia at HA mainly occurs in small pulmonary arteries. HAPE is often complicated by frostbite, trauma, infection, cerebral edema. It is commonly misdiagnosed as pneumonia.
Betz, 2015 [25]	Original research	42	They showed that some individuals with high PASP may protect them from HAPE as a consequence of high fluid reabsorption.
Li Y, 2018 [26]	Review	NA	They suggested that HAPE is caused by a combination of the following factors: pulmonary hypertension, sympathetic stimulation, defective NO synthesis, exaggerated endothelin-I synthesis, reduced fluid clearance from alveolar space, and pulmonary blood-gas barrier permeability elevation.

Clinical Relevance

**Figure 4:**
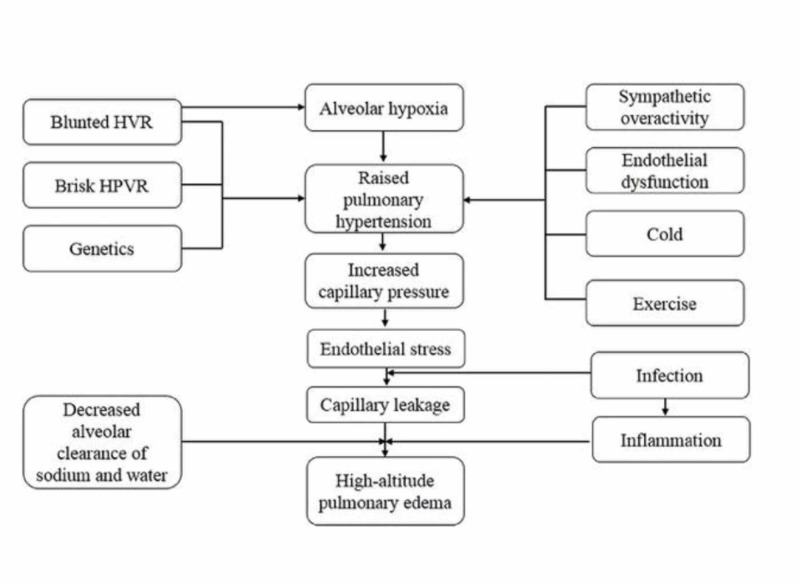
Proposed pathophysiology of high altitude pulmonary edema HVR: hypoxic ventilatory response, HPVR: hypoxic pulmonary vascular response Permission was obtained from the original publisher

HAPE is evident within one week of arrival above 2,500 to 3,000 m altitude [2]. Both well acclimatized high-altitude residents traveling from low altitude (known in this group as re-entry HAPE) and rapidly ascending non-acclimatized lowlanders can develop HAPE [2]. Major determinants in both groups are altitude, ascent rate, and individual susceptibility [2]. The incidence of HAPE increases from 1% to 10% when ascended rapidly at 4,559 m [22]. When a person is subjected to a hypoxic environment at high altitude (HA), peripheral chemoreceptors in the carotid bodies detect the change in the partial pressure of oxygen in the blood [27]. As a result, acute hypoxic ventilatory response (AHVR) is initiated by glomus cells of carotid bodies through the process of detection and transduction [27]. Hypocapnia and HPV are initiated due to AHVR in pulmonary arterioles (PAs) [27]. The adaptive response of HPV optimizes ventilation-perfusion matching and gas exchange. When this adaptive response is prolonged, it can result in HAPE [14]. Ventilatory acclimatization occurs in a distinct pattern in response to hypoxia; fast stimulation then declines within minutes, finally increases in ventilation over the next few hours to days [6]. Initially, there is an excessive drop in arterial saturation as a result of reduced static lung volumes, weakness of inspiratory muscles, and impaired diffusing capacity [23]. Sympathetic stimulation, pronounced nocturnal hypoxemia, instability in ventilatory control due to reduced lung compliance and impaired gas exchange are signs preceding overt HAPE [4]. There is interstitial fluid accumulation as a consequence of increased tidal volume and nocturnal breath rate [23]. Bailey, et al. described the association between free radical-mediated reduction of pulmonary nitric oxide (NO) bioavailability and increased pulmonary artery systolic pressure (PASP) at HA [14]. Apart from NO, carbon monoxide (CO) and hydrogen sulfide (H2S) are considered to be modulators of HAPE [2]. At the cellular level, reactive oxygen species (ROS) formation in mitochondria has been postulated as a primary stimulus for HPV [14]. Basnyat, et al. proposed the pathophysiology of HAPE shown in Figure 4 [28].

Classification of HAPE according to severity based on different parameters presented by Hackett and Roach is explained in Table 2 [29].

**Table 2:** Hackett and Roch classification of high altitude pulmonary edema

Classification	Symptoms	Heart rate/min	Respiratory rate/min	X-ray findings
Mild	Dyspnea on moderate exertion	110	20	Opacities involving <1/4 of 1 lung field
Moderate	Dyspnea, weakness, and fatigue on slight exertion Inability to perform light task or headache with cough or dyspnea at rest.	110-120	20-30	Opacities involving at least 1/2 of 1 lung field.
Severe	Severe dyspnea, headache, weakness and nausea at rest, productive cough, wheezy difficult respiration, and apparent cyanosis	120	30	Opacities involving at least 1/2 of each lung field or unilateral infiltrates involving an entire lung field

Hypoxic Pulmonary Vasoconstriction

Swenson, et al. suggested increased microvascular pressures with pulmonary arterial hypertension as a crucial factor in the pathophysiology of HAPE rather than upstream arterial pressure elevation [2]. The following three mechanisms were proposed in their study leading to edema formation due to hypoxic arteriolar constriction:

- Diffuse regional vasoconstriction

- Transarteriolar leak

- Hypoxic venoconstriction

Diffuse regional vasoconstriction leading to the heterogeneous nature of alveolar edema is ascertained by an asymmetric distribution of rales and infiltrates in clinical examination during the early phase of edema [24]. Alveolar leak predominantly occurs in areas with high blood flow as a result of less HPV [24]. The collected fluid is non-inflammatory and hemorrhagic which may secondarily evoke inflammatory response [24]. Alveolar epithelial Na- channels (ENaC), basolateral Na/K- ATPase, and chloride transport helps to clear alveolar space fluid osmotically to maintain electroneutrality [25]. If there is impaired fluid clearance, oxygen diffusion across the alveolar barrier is impaired causing hypoxemia and HPV [25]. Reduced alveolar fluid clearance by both type I and type II pneumocytes contributes to the pathophysiology of HAPE [26]. 

Mean PASP and HPV are higher in HAPE susceptible (HAPE-S) subjects compared to HAPE resistant (HAPE-R) group, however, the mechanism is not completely understood and is believed to be multifactorial [2, 22]. Following parameters are commonly implicated in HAPE-S subjects [2]:

1. Hemodynamics 

 - Exaggerated HPV

 - Pronounced pulmonary artery (PA) pressure elevation and sympathetic tone elevation

 - Decreased NO production.

 2. Pulmonary

 - Reduced diffusion capacity, alveolar Na/H2O reabsorptive capacity, and lung volumes.

 3. Ventilation and renal

 - Less hypoxia-induced hypoxic ventilatory responsiveness (HVR) due to reduced natriuretic response

Inflammation

Sharma, et al. described up-regulation of transcripts of inflammation in the body of HAPE subjects. Such transcripts result in a positive feedforward loop of endothelial permeability and fluid leakage through altered gap junction expression and endothelial activation. They also demonstrated increased TNF a, IL-6, VEGF, and NO in HAPE subjects that increase permeability in the lungs [30]. Ahmad, et al. identified major alterations in the expression of newly identified proteins involved in inflammation, immunity, oxidative stress, hemostasis, and signaling in hypoxic lungs [31]. Hilty, et al. presented that elevated soluble urokinase-type plasminogen activator receptor (SuPAR) to be a marker of HAPE susceptibility [15]. Similarly, they described increased levels of CRP and IL-6 plasma concentrations owing to hypoxia. However, they concluded HAPE to be modulated but not caused by inflammatory processes [15]. Ren, et al. found that tissue plasminogen activator (tPA), D- dimers, fibrinogens, and fibrin degradation products (FDPs) levels are increased due to hypoxia at HA [32]. They suggested an important factor of HAPE to be disequilibrium between coagulation and fibrinolysis resulting in lung injury [32]. However, Swenson, et al. in their study compared numerous literature and pointed out that only IL-6 to be elevated in both systemic circulation and alveolar space (elevated only after the onset of HAPE) [2]. As IL-6 can also be produced from exercising muscles, driven by the sympathetic nervous system, it may probably be the body’s effort to limit inflammatory damage and capillary permeability [2]. The following articles discussed the role of potential inflammatory mediators in the pathogenesis of HAPE (Table 3).

**Table 3:** Articles discussing inflammation as a pathophysiological basis of HAPE HAPE: high altitude pulmonary edema; TNF-a: tumor necrosis factor-alpha, VEGF: vascular endothelial growth factor, IL: interleukin, SULT1A1: sulfotransferase 1A1, SuPAR: soluble urokinase-type plasminogen activator receptor, CRP: C-reactive protein, HA: high altitude

First author, Year	Design of study	Total number of subjects	Main points
Ren, 2012 [32]	Clinical Trial	61	All patients in this study developed HAPE within 48 hours after ascent by airplane to an altitude of 3600 m in Tibet. To avoid activation of coagulation, blood samples were collected without tourniquets and negative suction within 30 minutes after admission and at the time of recovery. Fibrinolysis and coagulation systems are deranged in HAPE and this derangement is directly proportional to the severity of HAPE. Disequilibrium between coagulation and fibrinolysis results in lung injury. In some fatal cases, clots are found obstructing pulmonary blood vasculature.
Sharma, 2014 [30]	Clinical Trial	31	They showed the interaction of multiple pathways regulating vascular homeostasis and regulation of HAPE by multiple genes. They described the up-regulation of transcripts involved in inflammation and demonstrated increased TNF a, IL-6, VEGF, and NO in HAPE subjects.
Ahmad, 2015 [31]	Clinical Trial	20	They described the altered expression of newly identified proteins involved in inflammation, immunity, oxidative stress, homeostasis, and signaling in hypoxic lungs. For the first time, SULT1A1, a novel biomarker for diagnosis of HAPE is described in their study.
Hilty, 2016 [15]	RCT	41	Inflammation sets in at HA during rapid ascent. They described SuPAR to be a marker of HAPE susceptibility. Hypoxia increases levels of CRP and IL-6 in plasma of HAPE susceptible individuals.

Genetics

Recently, much attention has been given to acquire knowledge on the genetic basis of HAPE [10]. When an individual has previous experience of HAPE, the prevalence of having such episodes in the future is highly increased (>60%) in high-altitudes indicating individual susceptibility and genetic basis of HAPE [6]. The articles presented in Table 4 discussed the genetic alteration and variation as the pathophysiology of HAPE.

**Table 4:** Articles discussing the genetic basis of HAPE HAPE: high altitude pulmonary edema; EGLN1: encoding HIF (hypoxia-inducible factor)-prolyl hydroxylase 2, SaO2: oxygen saturation, RAAS: renin-angiotensin-aldosterone-system, PASMC: pulmonary arterial smooth muscle cells, ROS: reactive oxygen species, BMP: bone morphogenetic protein, ALK- 1: activin receptor-like kinase-1, BMPR-2: bone morphogenetic protein receptor-2, 5-HTT: 5-hydroxytryptamine transporter (serotonin transporter); VSMCs: vascular smooth muscle cells; ECs: endothelial cells; RNA: ribonucleic acid; DNA: deoxyribonucleic acid

First author, Year	Design of study	Total number of subjects	Main points
Mishra, 2013 [33]	Clinical Trial	890	EGLN1, an actual oxygen sensor, is inversely correlated with SaO2 levels. In the hypobaric hypoxic environment, EGLN1 has a potential role in functional adaptation.
Mishra, 2015 [27]	Review	NA	Responses involved in the pathogenesis of HAPE include alterations in pathways such as oxygen sensing, hypoxia signaling, calcium and potassium-gated channels, RAAS, and oxidative stress. Gene regulation by small RNAs, histone modification and DNA methylation predispose susceptible subjects to HAPE. Pulmonary endothelial cells (ECs) and vascular smooth muscle cells (VSMCs) are significantly affected due to hypoxia-induced impairment of voltage-gated (KV) channel resulting in vasoconstriction, PASMC contraction, medial hypertrophy, and hyperplasia. ROS formation and reduced antioxidants disrupt homeostasis, damages lipids, proteins, and DNA.
Ali, 2016 [10]	case-control; clinical trial	400	Together with elevated BMP-2 and 5-HT (5-Hydroxytryptamine) levels, there are genetic interactions among ALK-1, BMPR-2, and 5-HTT polymorphisms. This leads to differential gene expression contributing to the genetic basis of HAPE pathophysiology.

Markers of HAPE

Recently many markers have been identified to determine HAPE susceptibility. Some of the markers that can be used are elevated BNP (Brain Natriuretic Peptide) levels, asymmetric dimethylarginine (ADMA), 5-hydroxytryptamine (5-HT), and elevated hypoxia-inducible factor-alpha (HIF-a) [34, 35, 36].

Collectively, these data favor more on HPV as a pathophysiological basis of HAPE. But the inflammatory basis of HAPE pathophysiology is still a debatable issue as there are not many studies done at HA in HAPE patients to discard or validate this hypothesis with certainty. 

Limitations

This literature review has some limitations. We haven't included any literature older than 10 years, articles published other than English, and non-human studies. 

## Conclusions

HAPE is non-cardiogenic pulmonary edema occurring in rapidly ascending non-acclimatized healthy individuals. Our study mainly focusses on the pathophysiology of HAPE where hypoxia is a cause as well as consequence. Most of the literature favors diffuse patchy vasoconstriction as a result of hypoxia and sympathetic activation. However, some of the studies described inflammation in the lungs leading to HAPE. The genetic implication in HAPE can’t be overlooked as HAPE-S individuals have a higher propensity for HAPE. Resource-limited settings like HA have several difficulties to study subjects who develop as well as healthy individuals who are to develop signs and symptoms of HAPE. So, one of the future recommendations for researchers would be to develop large animal models that better mimics HAPE occurring in humans. As sympathetic activity is greatly increased with hypoxia, the role of the autonomic nervous system in HAPE should be explored. Artificially creating an ideal experimental environment that mimics HA conditions to study a large number of subjects would be another approach to gain more insights on the pathophysiology of HAPE.
